# Magnetic Lateral Flow Strip for the Detection of Cocaine in Urine by Naked Eyes and Smart Phone Camera

**DOI:** 10.3390/s17061286

**Published:** 2017-06-05

**Authors:** Jing Wu, Mingling Dong, Cheng Zhang, Yu Wang, Mengxia Xie, Yiping Chen

**Affiliations:** 1 Liver Research Center, Beijing Friendship Hospital, Capital Medical University, National Clinical Research Center of Digestive Diseases, 95 Yong-an Road, Xicheng District, Beijing 100050, China; wujing2013@mail.bnu.edu.cn (J.W.); dacheng1717@aliyun.com (C.Z.); 2 Analytical & Testing Center of Beijing Normal University, Beijing 100875, China; 3 CAS Key Laboratory for Biological Effects of Nanomaterials and Nanosafety, CAS Key Laboratory of Standardization and Measurement for Nanotechnology, CAS Center for Excellence in Nanoscience, National Center for Nanoscience and Technology, Beijing 100190, China; dongml@nanoctr.cn

**Keywords:** magnetic lateral flow strip, magnetic bead, smart phone camera, cocaine

## Abstract

Magnetic lateral flow strip (MLFS) based on magnetic bead (MB) and smart phone camera has been developed for quantitative detection of cocaine (CC) in urine samples. CC and CC-bovine serum albumin (CC-BSA) could competitively react with MB-antibody (MB-Ab) of CC on the surface of test line of MLFS. The color of MB-Ab conjugate on the test line relates to the concentration of target in the competition immunoassay format, which can be used as a visual signal. Furthermore, the color density of the MB-Ab conjugate can be transferred into digital signal (gray value) by a smart phone, which can be used as a quantitative signal. The linear detection range for CC is 5–500 ng/mL and the relative standard deviations are under 10%. The visual limit of detection was 5 ng/mL and the whole analysis time was within 10 min. The MLFS has been successfully employed for the detection of CC in urine samples without sample pre-treatment and the result is also agreed to that of enzyme-linked immunosorbent assay (ELISA). With the popularization of smart phone cameras, the MLFS has large potential in the detection of drug residues in virtue of its stability, speediness, and low-cost.

## 1. Introduction

Cocaine (CC) is obtained from the leaves of the coca plant which is considered as one of the most dangerous illegal drugs in last few decades. As a natural alkaloid with local anesthesia effect, CC has been widely used in the field of operative anesthesia at low doses. Meanwhile, CC is also a powerful nervous system stimulant, which may cause tremors, convulsions, and increased body temperature with excessive dosage. Widespread use and abuse of CC will cause serious social and health problems [1,2], hence, it is controlled internationally by the Single Convention on Narcotic Drugs. Therefore, it is very important to develop a rapid, low-cost, and reliable method for the detection of CC in blood, hair, or urine to ensure medication safety and drug control work, which could be further used in recording the history of CC abuse in the criminal detection. 

Currently, detection methods of CC mainly include instrumental analysis and immunoassay methods. Instrument analysis methods involve high performance liquid chromatography (HPLC) [3], liquid chromatography-mass spectrometry (LC-MS) [4,5], gas chromatography-mass spectrometry (GC-MS) [6,7], and capillary electrophoresis-mass spectrometry (CE-MS) [8], etc. These methods are highly sensitive and accurate. However, complicated pre-treatment and high cost have limited their application in point-of-care testing (POCT). Immunoassay methods mainly include enzyme-linked immunosorbent assay (ELISA) [5,9,10] and gold lateral flow strip (GLFS). ELISA has been commonly used in detecting drug residues in virtue of its specificity, sensitivity, and low cost. Nevertheless, ELISA is labor-intensive and time-consuming due to numerous wash steps [11]. GLFS is an alternative method for the detection of drug residues as its low cost, speediness, and ease-of-use. However, the sensitivity of GLFS is inferior to ELISA. Development of the LFS with a stable label probe for simple and rapid determination of CC with high sensitivity is strongly desirable. 

Up to date, magnetic bead (MB) has emerged and been applied in many aspects such as immuno enrichment and separation [12], magnetic sensing [13], drug carriers [14], magnetic resonance imaging (MRI) [15,16], and so on. MB is a multiple functional nanomaterial with its optical and magnetic properties, and it has been used as a novel signal probe in LFS for the detection of many targets [17,18,19]. This magnetic lateral flow strip (MLFS) not only retained the advantages of GLFS, such as low cost and fast detection, but also achieved quantitative determination when combined with a suitable magnetic detector, such as giant magnetoresistive effect sensor [17,20,21]. However, the cost of the system is largely increased with an auxiliary magnetic signal detector, which limits its application in developing countries. It is thus necessary to develop an alternative simplicity and low-coat MLFS platform to broaden its application in the point-of-care testing (POCT) field.

With the development of smart phone camera and image processing technology in the last three years, smart phone cameras have been used as a signal read out system to obtain a quantitative test resulting in the field of lateral flow strip (LFS) [22]. Owning to its convenient readout strategy and high popularizing rate, smart phone camera has been used as a powerful tool in the POCT field [22,23,24]. 

In this study, we present a novel MLFS based on a smart phone camera and image processing technology to realize qualitative and quantitative detection of CC in urine. The color of MB can be used as a visual signal, and the color density of MB can be transferred into gray value by image capturing software installed in a smart phone, which can be used as a quantitative signal. More importantly, MB is very stable at room temperature (RT), which is very useful for recording the history of drug abuse in criminal record.

## 2. Materials and Methods 

### 2.1. Chemicals and Apparatus

The 20, 30, and 150 nm magnetic bead (MB) (surface: COOH; saturation magnetization: 40 emu/g, 10 mg/mL) was obtained from Qcean NanoTech (San Diego, California, USA). The 30 nm gold nanoparticle (surface: COOH) was from Nanocs (New York, NY, USA). Bovine serum albumin (BSA), 1-ethyl-3-(3-dimethylaminopropyl)-carbodiimide hydrochloride (EDC), N-hydroxysulfosuccinimide sodium (NHS-S) were from Sigma-Aldrich (Saint Louis, MO, USA). Cocaine (CC, 900 ng/mL), morphine (Mop, 1000 ng/mL), amphetamine (Amp, 1000 ng/mL), Codeine (Cod, 1000 ng/mL), and CC monoclonal antibody were provided by Wondofo Biomedical Co., LTD (Guangzhou, China). The MACS separator with MS columns was from Micromod (Miltenyi Biotech Gmbh, Bergisch Gladbach, Germany). Millipore HF135 nitrocellulose (NC) membrane, absorbent pad, sample pad, polyvinyl chloride pad were purchased from Millipore (Billerica, MA, USA). 0.01M Tris buffer (pH = 8.0, 1% saccharose, 1% BSA and 0.02% NaN_3_) was used for developing solvent. All other chemicals were of analytical reagent grade and all the aqueous solutions were prepared with distilled water.

Supermag Separator (Qcean NanoTech, San Diego, CA, USA) was used for separation of 30 nm MB. XYZ3050-dimensional spray film analyzer (BioDot, Irvine, CA, USA) and CM4000 slitter (BioDot, Irvine, CA, USA) were employed for the preparation of MLFS strip. 3K30-high speed freezing centrifuge (Sigma, Osterode am Harz, Germany) and MS3 vortex oscillator (IKA Inc., Staufen, Germany) were employed to prepare MB-antibody (MB-Ab) conjugate.

### 2.2. Preparation of MB-Ab Conjugate

First, 400 µL of MB (30 nm, 10 mg/mL) was suspended for 2 min. Then, 20 µL of EDC (50 mg/mL) and NHS-S (50 mg/mL) were added to the MB solution. After activation for about 20 min, the excessive EDC, NHS-S and the byproducts were removed via magnetic separation using the MACS column. Subsequently, 0.2 mg antibody (Ab) was added to the activated MB solution, and the mixture was gently stirred to react for 2 h at RT. After that, the unconjugated Ab was removed using MACS column again. Finally, the solution of MB-Ab was redissolved in PBS (0.1% BSA) at 4 °C.

### 2.3. Preparation of MLFS 

The strip consisted of three components: a sample pad, a nitrocellulose (NC) membrane and an absorbent pad. All of these three components were attached onto a polyvinyl chloride (PVC) pad. Goat anti-mouse IgG (2.0 mg /mL) and CC-BSA conjugate (1.0 mg/mL) were separately sprayed onto the surface of NC membrane to form a control (C) line and a test (T) line, respectively. The sample pad was treated with PBS containing 1% BSA for 30 min, and they were dried at 37 °C for 2 h. Finally, this MLFS was stored at room temperature (RT). The lengths of sample pad, NC membrane and absorbent pad were 2.2, 1.7, and 2.0 cm. The width of the MLFS was 0.2 cm. The distance of T line and C line was 0.5 cm. 

### 2.4. The Process of MLFS for Detection of CC

The mixture solution of 80 µL of sample solution containing CC and 20 µL of MB-Ab in developing solvent was applied onto the surface of sample pad. The presence or absence of brown color in the T line could be judged as qualitative signal captured by a smart phone camera within 10 min. The obtained picture was processed into gray value by using the homemade image software. 

### 2.5. The Sensitivity of MLFS

Different concentrations of CC (1000, 500, 250, 100, 50, 10, 5, 2, 1, 0 ng/mL) were used as competitive antigen to obtain optimum sensitivity. Each sample was assayed thrice (n = 3). Calculation was performed using the OriginPro8.0 software. 

### 2.6. The Specificity of MLFS

Morphine (Mop), amphetamine (Amp), and codeine (Cod)—whose molecular structures are similar to CC and considered as CC’s structural analogs—were used to evaluate the specificity of the MLFS. The concentration of CC and its structural analogs were 50 and 500 ng/mL, respectively. 

### 2.7. Real Sample Analysis

Eight of CC urine samples from gowsters were provided by Wondofo Biomedical Co., Ltd. (Guangzhou, China). These urine samples have no pretreatment and were stored at 4 °C for further analysis. 

## 3. Results and Discussion

### 3.1. The Principle of MLFS

**Scheme 1:**
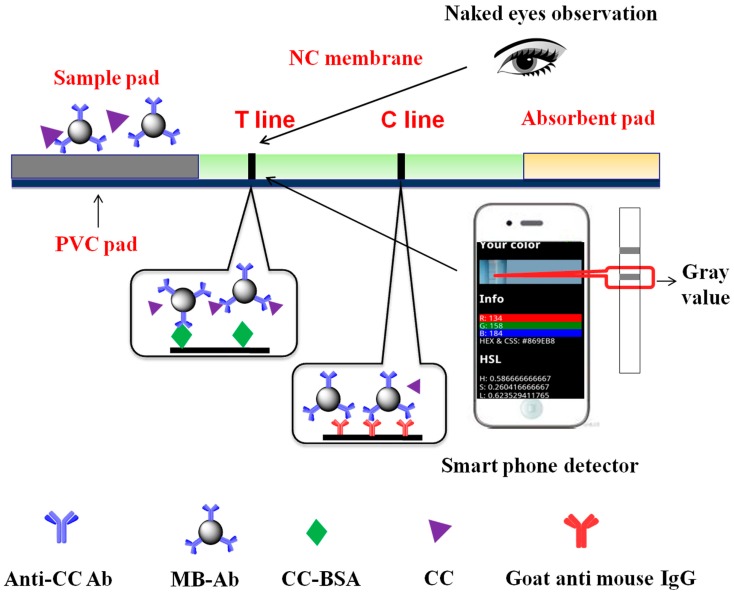
The principle of MLFS for detection of CC based on smart phone camera. Sample of CC and MB-Ab are applied to the sample pad and migrates along the strip, CC and CC-BSA competitively react with MB-Ab on the T line. The complex of CC with MB-Ab and the excess MB-Ab migrate along the membrane and are captured by the coated goat anti-mouse IgG on the C line. The color densities of the MB-Ab conjugate on T and C lines are measured by a smart phone (color scanner, weixun lin, China).

In this study, MB was chosen as the labeling material to develop a lateral flow strip based on competitive immunoassay format for detection of CC (Scheme 1). In this competitive immunoassay assay, free CC in the sample competed with the BSA-CC conjugate immobilized on the surface of T line to react with the MB-Ab conjugate. When there was no CC in the sample solution, the MB-Ab conjugate completely reacted with the immobilized CC–BSA, resulting in a darker color density of T line than that of the CC sample solutions. The excess MB-Ab or MB-Ab-CC complex could then react with goat anti mouse IgG on the C line. Thus, two bands appeared due to the accumulation of brown-colored MB-Ab conjugate. Furthermore, the color density of MB in T and C lines can be transferred into a digital signal by image capturing software installed in smart phone, which can be used as a quantitative signal.

### 3.2. Optimal Detection Conditions

Many factors affect the property of MLFS, the size of MB and the amount of MB-Ab are the two factors should be optimized. 

**Figure 1:**
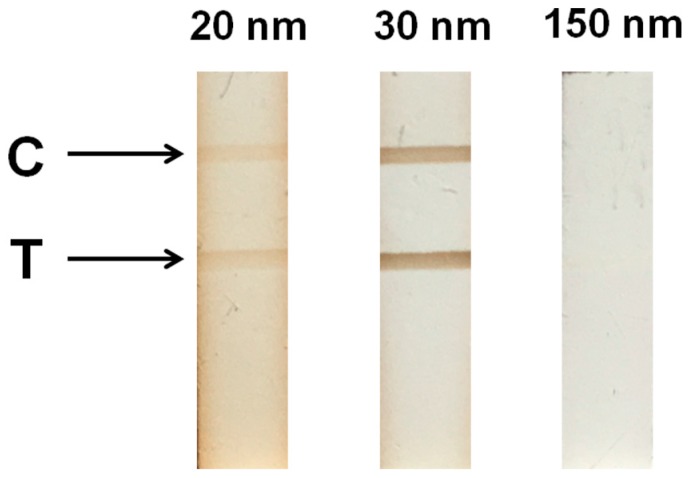
Optimization of the size of MB for detection of CC sample. Different sizes of MB-Ab (20, 30, and 150 nm) are prepared to detect the blank sample. The concentrations of the MB-Ab with different sizes are all 10 mg/mL.

For investigation of the optimal size of MB for detection of CC, three different sizes (20, 30, and 150 nm) of MB were prepared to detect the blank sample (Figure 1). 20 and 30 nm MB can evenly migrate NC membrane from bottom to top, and the whole analysis time only required 10 min, which were much more suitable for the application of POCT. Furthermore, the color densities of 30 nm MB-Ab on the T and C lines were much deeper than those of 20 nm MB. Moreover, the color of the background for 20 nm MB-Ab on the NC membrane was much deeper than those of 30 nm and 150 nm MB-Ab. We speculated that due to the too small size of 20 nm MB-Ab, some of MB-Ab deposited in the cracks of membrane resulting in a higher background than others. Moreover, the coupling efficiency for Ab and the stability of 20 nm MB were lower than those of 30 nm MB, which resulting in the better performance of 30 nm MB upon 20 nm. When using 150 nm MB-Ab, a large part of MB did not migrate to the top of NC membrane and aggregated on the sample pad (Figure S1), which affected the readout. Therefore, 30 nm MB was chosen as the magnetic probe in the strip for next experiments.

Further, we optimized the volume of MB-Ab for detection of CC sample with different conditions (10, 20, 40, and 60 μL) (Figure S2). The color densities of both T and C lines increased with the increasing volume of MB-Ab. In addition, when the volume of MB-Ab increased more than 20 μL, the background on the NC membrane became more obvious (Figure S2a). The darker color density will result in the lower gray value. It was thus that the gray value ratio of T/C decreased with the increasing volume of MB-Ab (Figure S2b). In addition, there was no obvious difference between the gray value ratios of MB-Ab with 20, 40, 60 μL. In view of these previous advantages, 20 µL MB-Ab was chosen as the best volume for subsequent experiments.

### 3.3. The Sensitivity of MLFS

**Figure 2:**
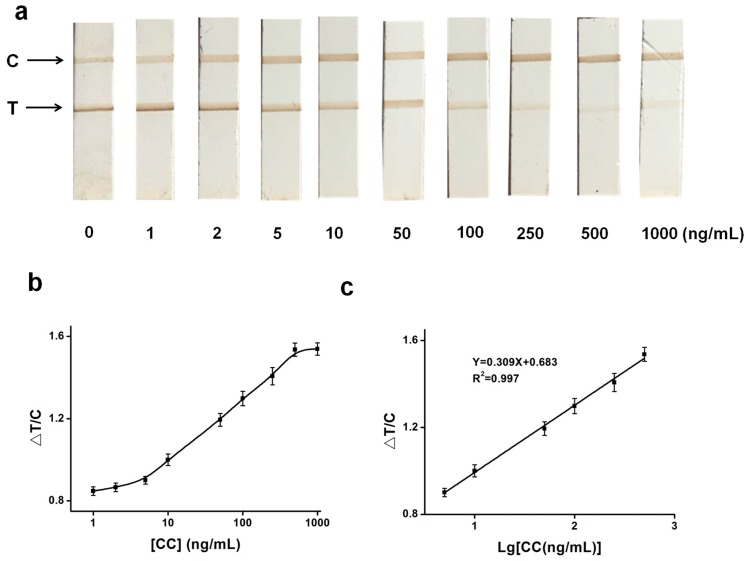
The sensitivity of MLFS for detection of CC samples in PBS. (**a**) Different concentrations of CC (1–1000 ng/mL) are detected by MLFS with the competitive immunoassay format. (**b**) The relationship of △T/C gray value with the concentration of CC is constructed. (**c**) The quantitative curve for detection of CC ranges from 5 to 500 ng/mL. The error bars represent the standard deviation from the three repeats (n = 3).

Under the optimal conditions above, a series of CC standard solutions in PBS solution were supplied on the CC strips to investigate the sensitivity of MLFS (Figure 2). A visual decrease in the intensity of color in the T line was obtained with increasing CC concentrations (1–1000 ng/mL) (Figure 2a). When the concentration of CC was 5 ng/mL, the color density of MB-Ab was slightly weaker than that of the blank. However, there was no difference about the color of MB-Ab between 2 ng/mL and blank sample, then the visual LOD was determined to be 5 ng/mL. The quantitative results for the detection of CC were also obtained based on the relationship of △T/C and the concentration of CC (T/C represented the ratio of the gray value of T line to that of C line, △T/C was obtained from the sample value of T/C minus that of the blank) (Figure 2b). △T/C for the MB-Ab’s color increased with the increasing concentration of CC from 0 to 1000 ng/mL (the gray value decreased with the increasing color density of MB-Ab). The LOD for quantitative detection of MLFS was determined based on the following formula: LOD = 3S/M, in which S was the value of the standard deviation of blank sample (the lowest concentration with the minimum response as the blank sample), and M was the slope of standard curve within the linear range of the low concentration. Therefore, the LOD of this MLFS using smart phone camera was 2.5 ng/mL (S = 0.011, M = 0.013). The linear detection range was 5–500 ng/mL, and the linear equation was Y = 0.309X + 0.683 (X = Lg [CC], R^2^ = 0.997), which can fully meet the current requirements for rapid screening of CC (Figure 2c). Furthermore, the RSD at the different spiked concentrations of CC was below 10%, which showed that this strip has good repeatability. For comParison, GLFS was also applied for CC detection with 30 nm of gold nanoprticle (The preparation for gold nanoparticle-Ab was based on previous report of our group [25]). The visual and quantitative LOD of GLFS were 20 ng/mL and 3.75 ng/mL (S = 0.02, M = 0.016), respectively, which were higher than those of MLFS (Figure S3). With the popularization of the smart phone camera, the MLFS combined with smart phone camera will be more convenient for operation, which can significantly broaden its application in drug detection. Furthermore, with the very stable chemical property of MB, the stability of MLFS is much better than other biotechnological detective method. Therefore, it is a useful method for the detection of drugs and recording the related information for long time, which is very important in the field of criminal investigation.

### 3.4. The Specificity of MLFS

**Figure 3:**
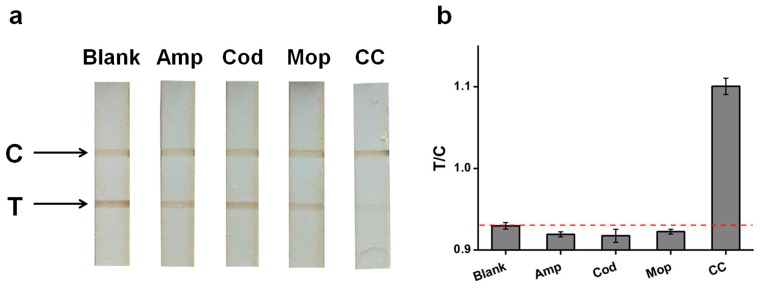
The specificity of MLFS for detection of CC samples. (**a**) 50 ng/mL of CC and the analogs such as Mop, Amp, Cod are detected using MLFS. The concentrations of the analogs are all 500 ng/mL. The negative sample is used as the blank. (**b**) The comparison of the T/C between the analogs and the CC sample. The error bars represent the standard deviation from the three repeats (n = 3).

Based on the discussion of the system’s sensitivity, the selectivity of MLFS was also investigated (Figure 3). The analogs such as morphine (Mop), amphetamine (Amp), codeine (Cod) were used to evaluate the selectivity of MLFS. Compared to CC, the color densities of MB-Ab on the T lines were much deeper when the concentrations of the analogs were 500 ng/mL, and the solution without CC was used as the blank (Figure 3a). In addition, the gray value ratios of T/C for the analogs were all lower than that of the blank (Figure 3b). Thus, we concluded that the MLFS has good specificity for detection of CC.

### 3.5. Real Samples Analysis

**Figure 4:**
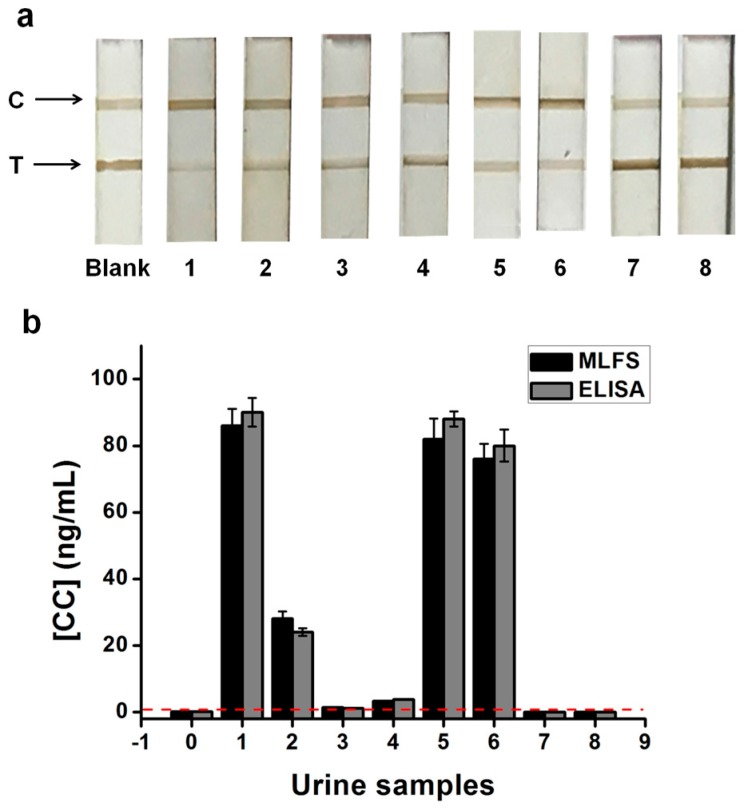
Real sample analysis of MLFS for CC urine samples. (**a**) Eight urine samples are detected and compared with the blank sample. (**b**) The comParison of MLFS and ELISA for quantitative detection of CC urine samples. The red dashed line represents the concentration of CC in blank sample. The error bars represent the standard deviation from three assays (n = 3).

To illustrate the potential application of the presented MLFS in practical samples, eight urine samples were collected and used for CC detection with this MLFS, and one negative urine sample was used as the control (Figure 4). Urine samples were directly dropped on MLFS without further pre-treatment. The color densities of the T lines for three samples (samples 1, 5 and 6) were much lower than that of the blank sample, suggesting that these samples were positive samples (Figure 4a). The color densities of T lines for samples 7 and 8 were significantly deeper than others and similar to that of the blank, which suggesting that the two samples may be negative. In addition, their concentrations obtained from the standard curve were 0.043 and 0.041 ng/mL, which were lower than that of the blank (0.05 ng/mL) (Table S1). These previous results demonstrated that samples 7 and 8 were the negative samples. The concentrations of CC samples were obtained from MLFS and ELISA (Figure 4b and Table S1). The comparison between MLFS and ELISA for quantification of CC samples showed a good agreement of two methods (Figure 4b) and the relative errors between the results of MLFS and ELISA were all under 20% (Table S1). The previous results suggested that the developed MLFS combined with a smart phone camera has great accuracy and selectivity, which can be applied to detect CC in real urine samples.

## 4. Conclusions

In summary, MLFS combined with a smart phone camera can simultaneously realize qualitative and quantitative detection of CC without increasing the analytical cost. Taking advantage of the high stability and sensitivity due to the high efficiency coupling for Ab through the covalent reaction (EDC/NHS activation), MLFS has improved the visual limit of detection to 5 ng/mL, which decreased four-fold compared to GLFS (20 ng/mL). Most importantly, the stability of MLFS is superior to that of GLFS, and the strip has been stored at room temperature for two years, which is suitable to record the history of drug abuse in the field of forensic testing. Furthermore, with the development of magnetic nanoparticles preparation and related imaging technology, it will provide a POCT platform for clinical diagnosis, food safety, environmental monitoring, and so on, and particularly in developing countries.

## Supplementary Materials

The following are available online at http://www.mdpi.com/1424-8220/17/6/1286/s1, Figure S1: Result for 150 nm of MB-Ab for detection of CC sample; Figure S2: Optimization of the volume of MB-Ab for detection of CC sample; Figure S3: The sensitivity of GLFS for CC detection in PBS; Table S1: Results of detection of CC urine samples by MLFS and ELISA.
