# Supplementary material for: Magnetic Lateral Flow Strip for the Detection of Cocaine in Urine by Naked Eyes and Smart Phone Camera
Source: Sensors (Basel). 2017 Jun 5;17(6):1286. doi: 10.3390/s17061286 (PMC5492392; doi:10.3390/s17061286)
Supplement: Supplementary file 1 [file sensors-17-01286-s001.pdf]

Magnetic lateral flow strip for the detection of cocaine in urine by naked eyes and smart phone camera

Jing Wu [1, 2], Mingling Dong [3], Cheng Zhang [1], Yu Wang [1*], Mengxia Xie [2*] and Yiping Chen [3,*]

[1] Beijing Institute for Tropical Medicine, Beijing Friendship Hospital, Capital Medical University, Beijing, 100050, China; dacheng1717@aliyun.com (C.Z.)
[2] Analytical & Testing Center of Beijing Normal University, Beijing 100875, China; wujing2013@mail.bnu.edu.cn (J.W.)
[3] CAS Key Laboratory for Biological Effects of Nanomaterials and Nanosafety, CAS Key Laboratory of Standardization and Measurement for Nanotechnology, CAS Center for Excellence in Nanoscience, National Center for Nanoscience and Technology, Beijing 100190, China; dongml@nanoctr.cn (M.L.D.)
* Correspondence: chenyp@nanoctr.cn (Y.P.C.); Tel.: 86-10-82545558; wangyuliver@126.com (Y.W.); Tel.: 86-10-63138435; xiemx@bnu.edu.cn (M.X.X.); Tel.: 86-10-58807981

Table S1 Results of detection of CC urine samples by MLFS and ELISA. Each sample was assayed thrice (n=3).

Urine samples	MLFS (ng mL^{-1})	ELISA (%)	Relative error (%)
Blank	0.05±0.001	0.048±0.001	4.1
1	86±5.1	90±4.3	4.4
2	28±2.2	24±1.1	17
3	1.3±0.15	1.1±0.05	18
4	3.2±0.22	3.8±0.12	16
5	82±6.1	88±2.3	6.8
6	76±4.5	80±4.8	5.0
7	0.043±0.001	0.045±0.001	4.4
8	0.041±0.001	0.044±0.001	6.8

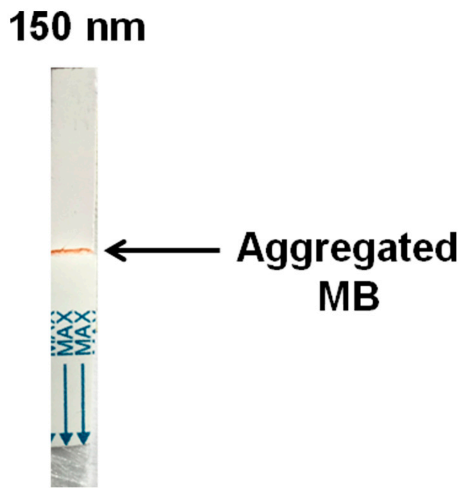

Figure S1 Result for 150 nm of MB-Ab for detection of CC sample.

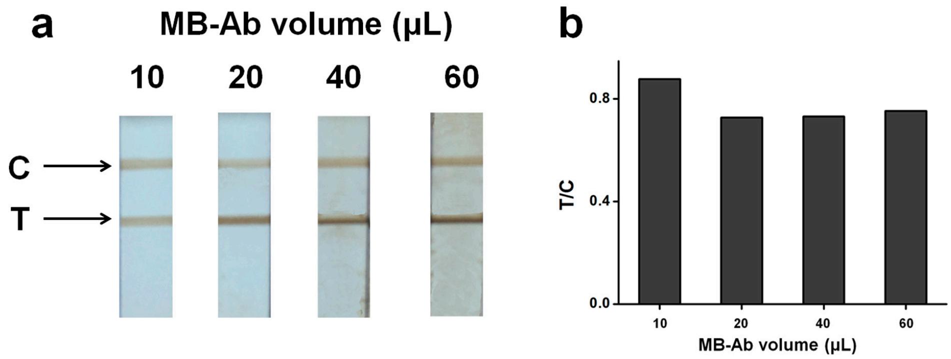

Figure S2 Optimization of the volume of MB-Ab for detection of CC sample. (**a**) Different volumes of 30 nm MB-Ab (10, 20, 40 and 60 μL) are prepared to detect the blank sample. (**b**) The gray value ratios of T zone/C zone (T/C) for the detection of the blank sample with different volumes of MB-Ab are obtained by smart phone camera. The concentration of the MB-Ab is 10 mg/mL.

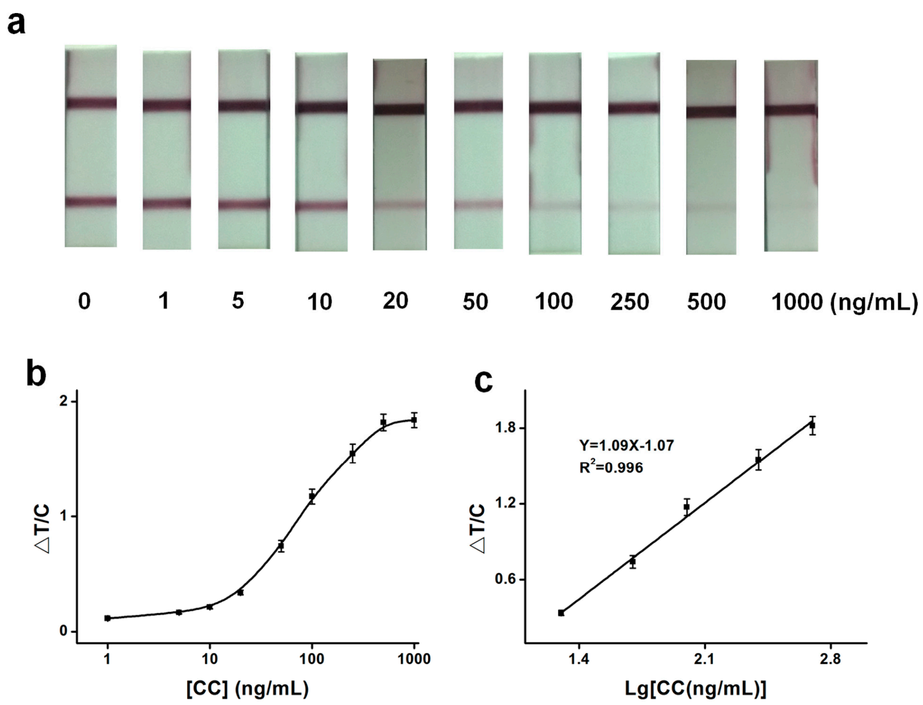

Figure S3 The sensitivity of GLFS for CC detection in PBS. (**a**) Different concentrations of CC (1-1000 ng/mL) are detected by GLFS with the competitive immunoassay format. (**b**) The relationship of ΔT/C gray value with the concentration of CC is constructed. (**c**) The quantitative curve for detection of CC ranges from 20 to 500 ng/mL. The error bars represent the standard deviation from the three repeats (n=3).
